# Book Reviews

**Published:** 1904-02

**Authors:** 


### BOOK REVIEWS.

A TEXTBOOK OF OBSTETRICS. By J. CLARENCE WEBSTER, M. D., (Edin.), Professor of Obstetrics and Gynecology, Rush Medical College, in Affiliation with the University of Chicago. Octavo, 767 pages, with 383 illustrations, 23 in colors. Philadelphia, New York, London: W. B. Saunders & Company. 1903. (Cloth, $5.00 net; sheep and half morocco, $6.00 net.)

To the already long list of excellent textbooks on obstetrics this one must be added. While, strictly speaking, we may not need another at present, yet it is quite within the bounds of propriety that the great field of obstetrics should be occupied by treatises written by competent teachers in every part of the country. A careful examination of this book justifies the opinion that the author is qualified not only to teach obstetrics, but also to write a textbook on the subject. His opportunities for observation in the great college where he teaches are almost unlimited, and he appears to have utilised them to advantage.

One of the best tests of the practical value of a work on obstetrics is the manner in which asepsis and antisepsis is applied,—a topic in which this treatise is not found wanting. It is pointed out, also, that this author has given prominence to anatomic changes in pregnancy, labor, and the puerperium, based largely on his own studies of frozen sections. Furthermore, he has presented embryologic, physiologic, and pathologic data that bear importantly upon many obstetric questions. These features serve to stamp the author's originality upon the work with marked emphasis. However, these researches, though minute in detail, have not prevented the proper unfolding of obstetrics in its clinical aspects. The student, whether undergraduate or junior practitioner, will always manifest special interest in the practical side of the subject, and will rely upon clinical teaching as affording the best instruction. It is at the bedside that he will acquire the art of diagnosis and treatment in its longest stride toward perfection. This treatise is the outgrowth of the highest recognition of this established principle. It teems throughout its pages with evidence of great clinical experience on the part of the author, who seems to know just how to interpret and apply this experience to the best advantage in teaching.

In the matter of illustrations the book is far beyond the average. Many of the pictures are original, and those which are not, have been taken from the most approved sources. As a final thought, we may add that as a practical textbook on obstetrics for both student and practitioner, there is left very little to be desired, it being as near perfection as any compact work that has been published.

---

A Treatise on Orthopedic Surgery. By Royal Whitman, M. D., Instructor in Orthopedic Surgery in the College of Physicians and Surgeons, Columbia University, New York. Second edition, revised and enlarged. Octavo, 820 pages, with 507 engravings, mostly original. Lea Brothers & Co., Philadelphia and New York. 1903. (Cloth, $5.50 net.)

Orthopedic surgery is a fascinating study to one who possesses mechanical skill and an aptitude for correcting deformities of the human body. Whitman has risen to the distinction of authority in this department of surgery, and has written a treatise that establishes his claim to high rank among those who teach and practise orthopedic surgery.

The first edition of the work met with favor and became exhausted sooner than was anticipated; this second one, therefore, is now put forth with such revisions as became necessary to bring it forward to the immediate present in its representations of this most important branch of medical science. In addition to other topics, perhaps quite as important, Whitman gives a complete dissertation of the Lorenz treatment of congenital dislocation of the hip-joint and other deformities, for which this famous surgeon has devised special methods of treatment. The Lorenz operation has attracted special attention of late, because of the visits of Lorenz to this country in 1902 and 1903, during which he demonstrated it at clinics held in the principal cities. It is, therefore, appropriate that it should be intelligently set forth in such a treatise by so competent an observer, for, indeed, Whitman himself was a participant in many of the clinics referred to.

The illustrations of the Lorenz operations are such exact reproductions that they convey nearly as good an idea of it as could be obtained by a personal attendance upon the clinics. Moreover, the entire book is especially well illustrated which adds to its general value. It is one of the best all-around working volumes of orthopedic surgery that has been printed.

A Textbook of Diseases of Women. By Barton Cooke Hirst, M. D., Professor of Obstetrics in the University of Pennsylvania; Gynecologist to the Howard, the Orthopedic, and the Philadelphia Hospitals. Octavo, 675 pages, with 650 mostly original illustrations, many in colors. Philadelphia, New York, London: W. B. Saunders & Co. 1903. (Cloth, $5.00 net; sheep or half morocco, $6.00 net.)

Based on a teaching and practising experience of twenty years Hirst offers this work to the profession as a gynecological guide, and adapts it to the average requirements of undergraduate, general practitioner, and specialist. He is not a stranger to the medical profession, his work on obstetrics issued several years ago, now in its fourth edition, having favorably introduced him.

The work begins with the usual instructions relating to examinations and local treatment, next considering anomalies of development in the general tract; then diseases and injuries of the vulva, including coccygodynia; after which follow in their regular order, diseases and injuries of the vagina; injuries and diseases of the cervix; displacements and diseases of the uterus; diseases of the endometrium, including disorders of menstruation and sterility; the fallopian tubes and extrauterine pregnancy; diseases of the ovaries; diseases of the pelvic connective tissue and of the peritoneum; diseases of the urinary tract; the detailed technic of gynecic surgery; and an index. It is arranged in parts, each of the foregoing titles constituting one part.

The author, as will be observed, has adopted the anatomical instead of the pathological classification, which he believes is the most logical as well as the most convenient arrangement of subjects. He also lays claim, and with reason, to having presented a concise description of the diseases of women in which special prominence is given to diagnosis and treatment. Furthermore, he has developed the technic of gynecic surgery in a most satisfactory and thorough-going manner, giving its minutest details, from the inception of anesthesia to the closure of the incision.

The illustrations are of a high order of merit. A large portion are from original photographs or water colors, and are presented in large numbers for the purpose of giving the text practical elucidation. Hirst's textbook of obstetrics has already taken its place in the first ranks as an authoritative exposition of the science and art of midwifery, and we have no doubt that this companion volume will take an equally high place in the literature of gynecology.

A Textbook of Pathology. By Alfred Stengel, M. D., Professor of Clinical Medicine in the University of Pennsylvania. Octavo volume of 933 pages, with 349 text-illustrations, many in colors, and 7 full-page colored plates. Fourth edition, revised. Philadelphia, New York, London: W. B. Saunders & Company. 1903. (Cloth, $5.00 net; sheep or half morocco, $6.00 net.)

The increasing interest in the study of pathology is indicated by the fact that the number of authors has multiplied within the last few years, until now there are numerous good textbooks offered to the student of morbid anatomy. Again, the book before us, first issued in 1898, has already reached its fourth edition,—a fact that would have been impossible twenty-five or thirty years ago. A further evidence of the growing concern of the profession in pathology is the fact that within even two or three years the accumulation of material has been embarrassing almost, and renders the task of revising a difficult one. Stengel, however, has sought to incorporate in this new edition only demonstrated facts, which renders his book all the more valuable.

The principle revision occurs in the section on general pathology, particularly Ehrlick's theories of immunity and allied processes, the chapters on inflammation, bacterial diseases,—especially typhoid fever, tuberculosis, yellow fever, and dysentery; also the section on diseases of the blood. Some new illustrations have been introduced, and a few have been eliminated which serves to freshen the treatise and keep it well to the fore in this respect. It is doubtful if the student of medicine today can find a more practical textbook on pathology.

---

A Textbook of the Practice of Medicine. By James M. Anders, M. D., Professor of the Practice of Medicine and of Clinical Medicine, Medico-Chirurgical College, Philadelphia. Sixth edition, thoroughly revised. Octavo, 1,300 pages, fully illustrated. Philadelphia, New York, London: W. B. Saunders & Company. 1903. (Cloth, $5.50 net; sheep or half morocco, $6.50 net.)

So recently have we commented on the excellence of Anders's practice, not once nor twice but several times as each succeeding volume has appeared, that we feel it almost the work of supererogation to further discuss its claims to professional favor. However, it is proper to say that there are a number of topics that belong to the changing and rapidly growing class, as far as our knowledge of them is concerned. The author has considered all these subjects in the new edition, among which are mosquito-borne diseases, bacillary dysentery, cholecystitis, certain animal parasitic diseases, the use of the *x*-rays in diagnosis and treatment; also several new subjects have been introduced, among which are paratyphoid fever, the fourth disease, trypanosomiasis, orthostatic albuminuria, transcortical aphasia, adiposis dolorosa, and amaurotic family idiocy.

This treatise is preëminently a guide for the practising physician and student of medicine, and is unsurpassed as a textbook on internal medicine.

PROGRESSIVE MEDICINE. Fifth Annual Series. Volume IV. December, 1903. A Quarterly Digest of Advances, Discoveries and Improvements in the Medical and Surgical Sciences. Edited by HOBART AMORY HARE, M. D., Professor of Therapeutics and Materia Medica in the Jefferson Medical College of Philadelphia. Octavo, 444 pages; illustrated. Philadelphia and New York: Lea Brothers & Co. (Per volume, $2.50, by express prepaid. Per annum, in four cloth-bound volumes, $10.00.)

The subjects dealt with in this volume are as follows: diseases of the digestive tract and allied organs, the liver, pancreas, and peritoneum, by John C. Hemmeter; anesthetics, fractures, dislocations, amputations, surgery of the extremities, and orthopedics, by Joseph C. Bloodgood; genitourinary diseases, by William T. Belfield; diseases of the kidneys, by John Rose Bradford; physiology, by Albert P. Brubaker; hygiene, by Charles Harrington; practical therapeutic referendum, by H. R. M. Landis.

One of the important features of this periodical is the excellent method of arranging and presenting the material. The headings and subheadings stand out in bold type and the printed page is agreeable to the eye. This makes it easy of reference and convenient for research. Moreover, the material itself comprises the better part of recent literature relating to the subjects set forth.

---

TRANSACTIONS OF THE TWENTY-FIFTH ANNUAL MEETING OF THE AMERICAN LARYNGOLOGICAL ASSOCIATION, held at Washington, D. C., May 12-14, 1903. James E. Newcomb, M. D., Secretary. New York: Rooney-Otten Co.

This volume of association transactions contains many interesting papers relating to diseases of the nose and throat. Dr. W. Scott Renner, of Buffalo, presented a paper entitled, Tertiary syphilis of the nose and pharynx, which is comprehensive in character and is a distinct addition to the literature of the subject. Dr. John O. Roe, of Rochester, read a paper, the subject of which is, The removal of obstructions and cicatricial contractions of the nose by the plastic method, which is well illustrated and contains many valuable suggestions, that contribute toward the further perfection of the surgery of the nasal passages. Many other interesting and useful subjects are discussed in this book which will prove of interest to every physician called upon to practise laryngology.

This association is one of the oldest of the special societies and is famous for the scientific work which it does at its annual meetings and is presented through its published transactions.

---

NOSE AND THROAT WORK FOR THE GENERAL PRACTITIONER. By GEORGE L. RICHARDS, M. D., Otologist and Laryngologist, Fall River Union Hospital, Fall River, Mass. Duodecimo, pp. 330. Illustrated. New York: International Journal of Surgery Co. 1903. (Price, $2.00.

A brochure constructed on lines similar to this book has long been needed. A general practitioner of medicine, with an aptitude for surgical manipulation and a knowledge of the anatomy and physiology of the nasal passages, can treat many of the simpler maladies of the nose and throat. Generally speaking, the larger textbooks are too elaborate and technical to serve as guides in diagnosis or treatment for any but specialists.

This author has prepared a little treatise that meets the want described. He gives enough anatomy and physiology to furbish up the memory and to lay the foundation for that which follows. In his description of instruments he only takes up such as are needed in ordinary diagnosis and treatment, the use of which can be readily learned by every intelligent physician. The illustrations have been chosen with special reference to the conditions named and if we mistake not, the book will serve a useful purpose. It is well made up, is of convenient size and reasonable in price.

---

THE PRACTICAL MEDICINE SERIES OF YEAR BOOKS. Ten volumes. Issued under the general editorial charge of GUSTAVUS P. HEAD, M. D., Professor of Laryngology and Rhinology in the Chicago Post-Graduate Medical School. Volume X. Skin and Venereal Diseases, Nervous and Mental Diseases. Edited By W. L. Baum, M. D., Hugh T. Patrick, M. D. Duodecimo, pp. 236. Chicago: The Year Book Publishers. 1903. (Price, $1.25; entire series, $7.50.)

The department of skin and venereal diseases, which comprises about one-half of this volume, is prepared by William L. Baum, professor of skin and venereal diseases in the Chicago Post-Graduate Medical School. Professor Baum has made discreet selection from the current literature of the past year and has compiled a most useful section of this volume. We note on page 94, an abstract of Dr. J. H. Dowd's paper in which he considered the use of sounds in urethral inflammation and for the dilatation of stricture.

The department of nervous and mental diseases, prepared by Hugh T. Patrick, professor of neurology in the Chicago Policlinic, and Charles L. Mix, professor of general medicine in the Post-Graduate Medical School of Chicago, presents a condensation of the literature of this branch of medicine that should prove valuable to the general practitioner.

Both departments contain several excellent illustrations which serve to amplify or explain the text in a satisfactory manner.

The Johns Hopkins Hospital Reports. Volume XI. Nos. 1-9. Baltimore: The Johns Hopkins Press. 1903.

The contents of this report includes a historical, clinical, and experimental study of pneumothorax, by Charles P. Emerson; clinical observations on blood pressure, by Henry Wireman Cook, and John Bradford Briggs, and a dissertation on the value of tuberculin in surgical diagnosis, by Martin B. Tinker. The first title covers the greater part of the volume,—450 pages,—and gives a summary of the literature from Hippocrates, fifth century B. C., to Avon, A. D. 1902,—358 references in all, which makes it a most valuable contribution for working purposes. The other papers are well prepared studies upon the topics which they respectively consider.

